# Differenzialdiagnosen des entzündlichen Hüftgelenks

**DOI:** 10.1007/s00117-021-00835-1

**Published:** 2021-03-25

**Authors:** Claudia Weidekamm, James Teh

**Affiliations:** 1 Universitätsklinik für Radiologie und Nuklearmedizin, Medizinische Universität Wien, Währinger Gürtel 18–20, 1090 Wien, Österreich; 2 Department of Radiology, Nuffield Orthopaedic Centre, Oxford University Hospitals NHS Trust, Windmill Road, Headington, OX3 7LD Oxford, Großbritannien

**Keywords:** Hüftschmerz, Magnetresonanztomographie, Entzündung, Diagnose, Coxarthrose, Hip pain, Magnetic resonance imaging, Inflammation, Diagnosis, Hip osteoarthritis

## Abstract

Die Differenzialdiagnosen der entzündlichen Hüfterkrankung spielen für die Diagnose des Hüftschmerzes eine bedeutende Rolle. Die rheumatologischen/entzündlichen Veränderungen des Hüftschmerzes mit dem entsprechenden Einsatz der Bildgebung wurden in Teil 1 des CME-Artikels abgehandelt. In diesem zweiten Teil wird ein systematischer Zugang erläutert, um die zahlreichen rheumatologischen Erkrankungen von Degenerationen, synovialen Tumoren und Infektionen zu unterscheiden. Die Interpretation der Pathologien in der Bildgebung im Zusammenhang mit dem klinischen Erscheinungsbild wird für die einzelnen Differenzialdiagnosen genauer erläutert. Das zeitgleiche Auftreten von unterschiedlichen Erkrankungen, zum Teil als sekundäre Komplikation, hat einen erheblichen Einfluss auf die Therapieplanung und sollte vom Radiologen erkannt werden.

## Abstract (en)

The differential diagnoses of inflammatory hip diseases play an important role for the diagnosis of hip pain. The radiological manifestations of rheumatic inflammatory hip pain with the corresponding use of imaging modalities were dealt in part 1 of the CME article. In this second part a systematic approach is explained in order to differentiate the many rheumatic diseaeses from degeneration, synovial tumors and infections. The interpretation of the pathologies seen in imaging in association with the clinical phenotype is more precisely elucidated for the individual differential diagnoses. The concomitant occurrence of different diseases, sometimes as secondary complications, has a substantial influence on treatment planning and should be recognized by radiologists.

## Lernziele

Nach Absolvieren dieser Fortbildungseinheit …

- verstehen Sie die Überlappung der entzündlichen Veränderungen von rheumatologischen Erkrankungen und ihrer Differenzialdiagnosen.
- können Sie die wichtigsten Differenzialdiagnosen zum entzündlichen Hüftschmerz identifizieren.
- können Sie die unterschiedlichen bildgebenden Verfahren in den diagnostischen Pfad zur korrekten Diagnose einsetzen.

## Einleitung

Der Hüftschmerz wird zumeist durch die Degeneration oder durch ein akutes Trauma verursacht. Im ersten Teil des CME-Artikels wurden die unterschiedlichen entzündlichen Erkrankungen, die einen Hüftschmerz verursachen, abgehandelt. Im zweiten Teil wird der Schwerpunkt auf die **Differenzialdiagnosen**

Differenzialdiagnosen

des entzündlichen Hüftschmerzes gesetzt. Das Spektrum der Differenzialdiagnosen zum Hüftschmerz ist weitgreifend, und es wird kein Anspruch auf Vollständigkeit in diesem Artikel erhoben. Allerdings werden die wichtigsten Differenzialdiagnosen besprochen, die unbedingt bei der Diagnosestellung berücksichtigt werden sollten. Eine Überlappung von Merkmalen unterschiedlicher Erkrankungen erschwert die eindeutige Diagnose in der Bildgebung sowie auch klinisch – dies umso mehr, wenn unterschiedliche Erkrankungen parallel oder als Folge voneinander auftreten und komplexe Pathologien vorliegen. Die wichtigsten 3 Merkmale der entzündlichen Arthritis, nämlich Knochenmarködem (KMÖ), Synovitis/Gelenkerguss und Erosionen, können auch bei anderen Krankheiten vorkommen (Tab. 1).

**Table 1:** 

Osteoarthritis
Septische Arthritis
Osteomyelitis
Riesenzelltumor der Sehnenscheide/pigmentierte villonoduläre Synovitis (PVNS)
Synoviale Chondromatose
Transientes Knochenmarködemsyndrom („transient BME [bone marrow edema]“)
Avaskuläre Nekrose (AVN)
Chondroblastom
Osteoidosteom

## Coxarthrose

**Figure 1:**
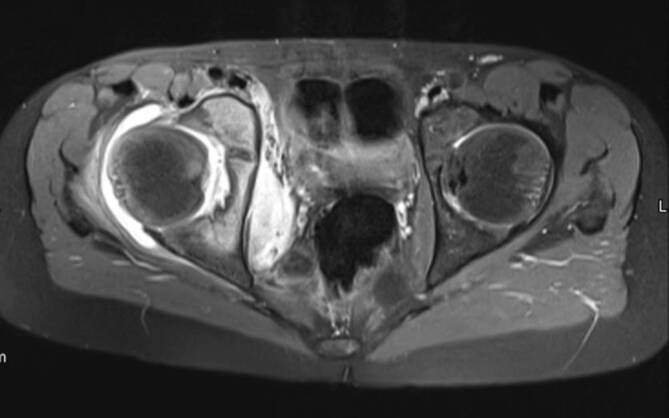

**Figure 2:**
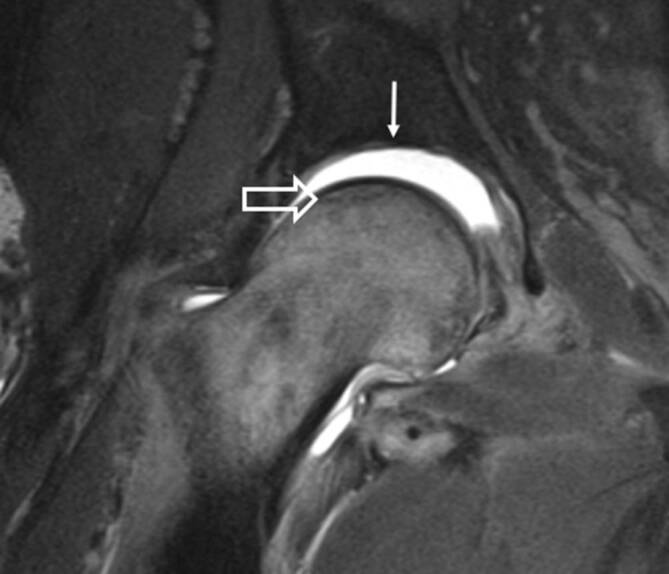

Die häufigste Indikation zur Bildgebung der Hüfte ist die Coxarthrose. Die Arthrose und die Entzündung der Hüfte haben einige Gemeinsamkeiten in der Bildgebung. KMÖ, Knorpelbelagverschmälerung, Synovitis und Gelenkerguss können bei beiden Entitäten vorkommen (Abb. 1). Hier sind einige Tipps, um die Coxarthrose von der Arthritis zu unterscheiden:

- Die Knorpelbelagverschmälerung bei der Arthritis ist diffus und betrifft die gesamte Gelenkfläche, hingegen ist bei der Degeneration typischerweise der Knorpel der gewichtstragenden Gelenkanteile verschmälert ([1]; Abb. 2). In seltenen Fällen kann es auch zu einer subchondralen Fraktur kommen (Abb. 2).
- Beim femoroazetabulären Impingement (FAI) vom Cam(engl.: „Nockenwelle“)-Typ, welches zu einer frühen Coxarthrose führen kann, befindet sich der Knorpelschaden im vorderen und superolateralen Anteil des Gelenks [2]. Beim Cam-Typ ähneln die Knochenanbauten am Oberschenkelhals einem Pistolengriff („pistol grip“), und gleichzeitig liegt eine asymmetrische Gelenkspaltverschmälerung vor.
- Bei der fortgeschrittenen Osteoarthrose zeigt sich in der Projektionsradiographie eine Gelenkspaltverschmälerung mit subchondraler Sklerose und Geröllzysten. Im Gegensatz dazu ist bei der rheumatoiden Arthritis (RA) eine gelenknahe Demineralisation anstatt der Sklerose zu sehen [3].
- Beim FAI vom Cam-Typ sind die subchondralen Zysten typischerweise am lateralen Rand des Azetabulums, angrenzend an den Knorpelschaden, zu finden. Das FAI vom Pincer-Typ ist durch eine überragende Überdachung („overcoverage“) des Femurkopfes charakterisiert, was durch eine Protrusio acetabulae oder durch marginale Osteophyten bedingt ist. Der Knorpelschaden ist hier typischerweise im posterioren Anteil des Azetabulums lokalisiert (selbst die Hochfeld-MRT [Magnetresonanztomographie] weist Limitationen in der Darstellung des Knorpelschadens bei der Früharthrose auf, da die Knorpelschichten der Hüfte im Allgemeinen zu dünn sind, um kleinere Defekte zu entdecken [4]).
- Spezielle Knorpelsequenzen und die Traktions-MR-Arthrographie verbessern die Darstellung von chondralen Delaminationsdefekten und oberflächlichen Knorpeldefekten [5, 6].

### Merke

Der Gelenkspalt ist bei der Coxarthrose im gewichtstragenden Gelenkanteil verschmälert, während bei der Arthritis der Gelenkspalt diffus verschmälert ist.

## Septische Arthritis/Osteomyelitis

**Figure 3:**
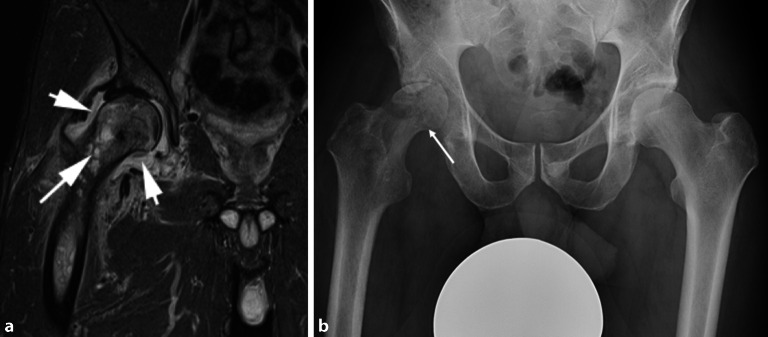

Eine verdickte und Kontrastmittel(KM)-anreichernde Gelenkkapsel lässt eine septische Arthritis vermuten. Wenn ein KMÖ nicht nur subchondral vorliegt, sondern in den Oberschenkelhals hineinreicht, dann besteht Verdacht auf eine Osteomyelitis (Abb. 3a). Im Frühstadium zeigen sich in der Projektionsradiographie eine Verdichtung des Fettgewebes und eine Vorwölbung der Gelenkkapsel, was auf einen **Erguss**

Erguss

hindeutet (Abb. 3b). Aber zumeist wird das Frühstadium einer Osteomyelitis in der Projektionsradiographie übersehen. Veränderungen wie Osteopenie, periostale Reaktion, fokale Lyse oder Diskontinuität des Kortex mit Knochendestruktion zeigen sich üblicherweise erst nach 5 bis 7 Tagen bei Kindern und nach 10 bis 14 Tagen bei Erwachsenen. Bei der subakuten oder chronischen Form der Osteomyelitis kann ein intramedullärer Abszess (**Brodie-Abszess**

Brodie-Abszess)

entstehen. Der Brodie-Abszess zeigt sich als eine lytische ovaläre Läsion in der Projektionsradiographie. In der Computertomographie (CT) erscheint der Abszess als zentral intramedulläre hypodense und zystisch erscheinende Läsion mit sklerotischer Begrenzung. In der MRT erscheint der Abszess mit einem pathognomonischen **Penumbra-Zeichen**

Penumbra-Zeichen

(T2-hyperintenser Granulationssaum), und die MRT ist die sensitivste und spezifischste Bildgebungsmodalität, um Gelenkkomplikationen zu identifizieren. Die **Antiobiotikatherapie**

Antiobiotikatherapie

ist häufig über 6 Wochen andauernd und nicht immer erfolgreich, was eine chirurgische Entfernung des Abszesses erfordern kann [7]. Im Vergleich zur nichtinfektiösen Entzündung ist die Beteiligung des periartikulären Weichteilgewebes bei der Infektion ausgedehnter (Abb. 3a). Die **Sonographie**

Sonographie

stellt Weichteilabszesse, subperiostale Flüssigkeitsansammlungen und assoziierte Gelenkergüsse dar und unterstützt die Aspiration von Gelenkergüssen und Flüssigkeitsansammlungen.

## Riesenzelltumor der Sehnenscheide

Der tenosynoviale Riesenzelltumor, auch **pigmentierte villonoduläre Synovitis (PVNS)**

**Figure 4:**
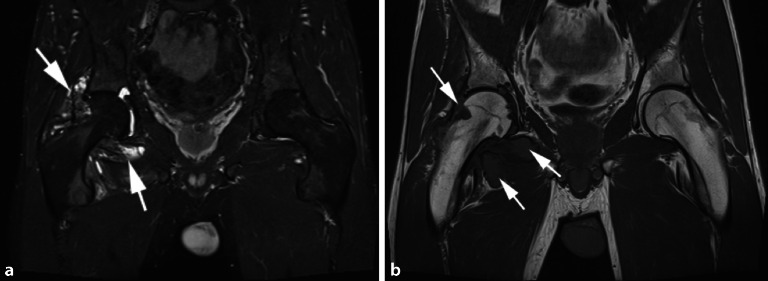

Pigmentierte villonoduläre Synovitis (PVNS)

genannt, ist ein lokal aggressiver Tumor, der aus der Synovia des Gelenks, der Bursa und der Sehnenscheide entsteht. Der Tumor kann lokalisiert oder in diffuser Form vorkommen. Das Kniegelenk ist mit etwa 80 % die häufigste Lokalisation für den Riesenzelltumor, jedoch kann auch die Hüfte in bis zu 16 % der Fälle betroffen sein. Typischerweise klagen die Patienten über Schmerzen und Gelenkdysfunktion. Die synoviale Proliferation ist nodulär und villös geformt, kann aber wie bei der entzündlichen Synovitis auch nur diffus verdickt erscheinen [9]. Die MRT spielt eine wichtige Rolle in der Diagnose des Riesenzelltumors, da die pathognomonischen Hämosiderinablagerungen in der T2-gewichteten Sequenz ein niedriges Signal aufweisen (Abb. 4a). In den Gradientenechosequenzen zeigen sich typische **Blooming-Artefakte**

**Figure 5:**
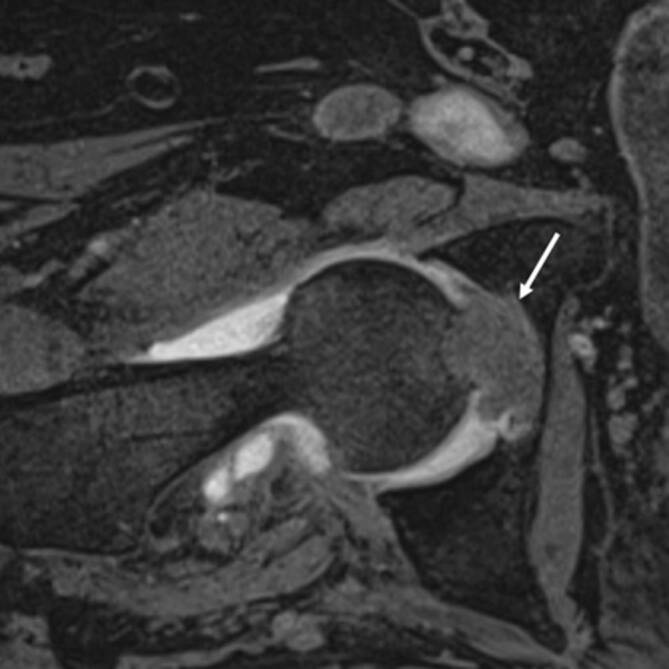

Blooming-Artefakte,

wodurch die Pathologie noch deutlicher auffällt. Erosionen können in manchen Fällen auftreten und sind teilweise an beiden Gelenkflächen bei regulärem Gelenkspalt nachweisbar (Abb. 4b). Die PVNS unterscheidet sich von einer fokalen Synovitis (Abb. 5) durch Erosionen und pathognomonische Hämosiderinablagerungen.

## Synoviale Chondromatose

Die synoviale Chondromatose kann in primärer oder sekundärer Form vorliegen. Die primäre synoviale Chondromatose (**Morbus Reichel**

**Figure 6:**
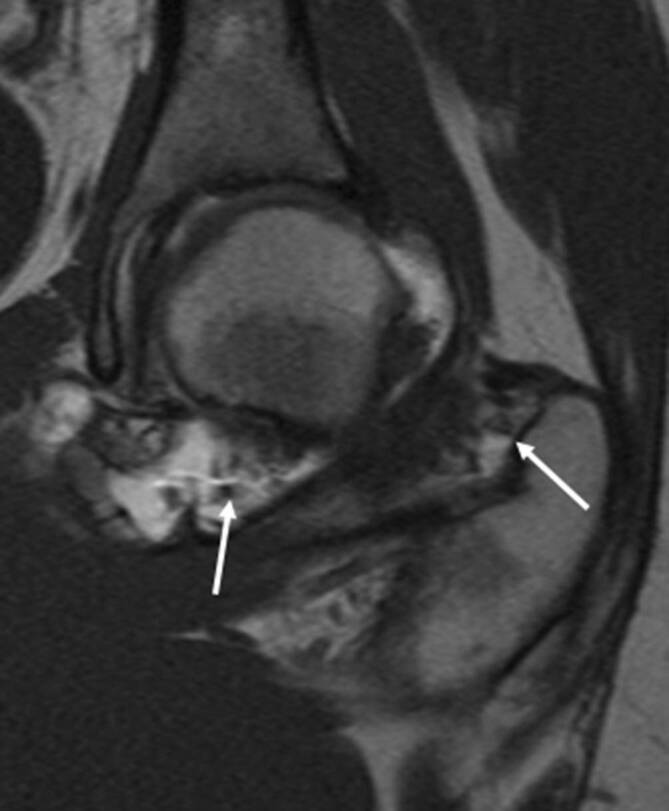

**Figure 7:**
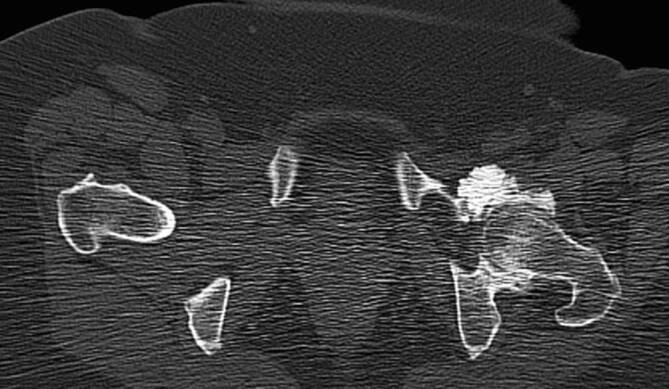

Morbus Reichel)

ist durch eine synoviale Metaplasie und zahlreiche intraartikuläre freie Gelenkkörper von ähnlicher Größe und runder Morphologie, meistens chondrogen, charakterisiert (Abb. 6). Die sekundäre Form weist typischerweise viel größere freie Gelenkkörper von unterschiedlicher Form und Größe auf [10]. Am häufigsten tritt die sekundäre synoviale Chondromatose bei fortgeschrittener Arthrose, Trauma oder neuropathischer Arthropathie auf. Die chondrogenen Körper können in der Synovia liegen oder sich davon ablösen und freie Gelenkkörper bilden, die mit der Zeit kalzifizieren oder sogar ossifizieren (Abb. 7). Das Ausmaß der Mineralisation ist ausschlaggebend, ob die Körper in der Projektionsradiographie oder in der CT sichtbar sind.

## Transientes Knochenmarködem/Osteoporose der Hüfte

**Figure 8:**
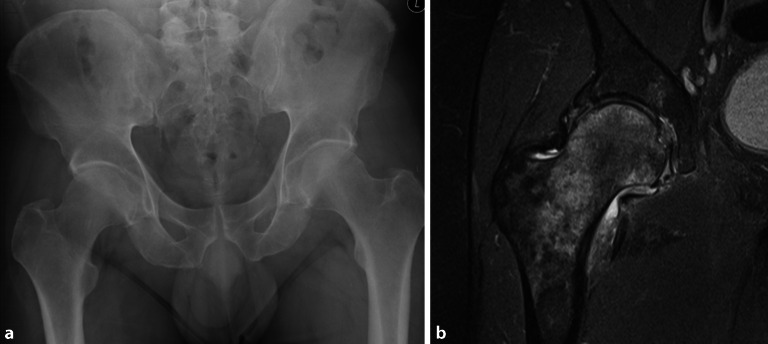

Das transiente KMÖ-Syndrom wird auch als transiente Osteoporose der Hüfte bezeichnet. Männer im 4. und 5. Lebensjahrzehnt und Frauen im 3. Trimester der Schwangerschaft sind typischerweise betroffen. Die Projektionsradiographie ist auffällig (Abb. 8a), weswegen die Diagnose üblicherweise in der MRT gestellt wird (Abb. 8b). Gewöhnlich liegt eine breite Übergangszone zwischen dem diffusen KMÖ und dem normalen Knochenmark vor (Abb. 8b). Das Ödem reicht üblicherweise in den subchondralen Knochen des Femurkopfes und des Femurhalses hinein, während die intertrochantäre Region und das Azetabulum seltener involviert sind [11]. Abgesehen vom KMÖ, ist der subchondrale Knochen regulär erhalten. Der häufig gleichzeitig auftretende Gelenkerguss ist vermutlich reaktiv. Die eindeutige Diagnose des transienten KMÖ-Syndroms wird in der Verlaufskontrolle gestellt, da die Krankheit selbstlimitierend ist und nach 6 bis 12 Monaten komplett ohne Komplikationen ausheilt. Klinisch zeigt sich ein spontaner Schmerz der Hüfte ohne vorausgegangene Ursache oder pathologische Rheumaserologie.

## Avaskuläre Nekrose

Die avaskuläre Nekrose (AVN) ist ein pathologischer Prozess, der aus einer Unterbrechung der Gefäßversorgung für den Knochen resultiert. Die **ischämische Zirkulationsstörung**

Ischämische Zirkulationsstörung

kann durch verschiedene Pathomechanismen wie Störung der arteriellen Zufuhr oder des venösen Abflusses, Verlegung der intraossären Kapillaren und intraossäre Gefäßkompression durch extravaskuläre Faktoren entstehen, die allesamt zum Zelltod des Knochenmarks und in weiterer Folge zu subchondralen Frakturen und zu einem Einbruch des Femurkopfes führen. Das Endstadium ist eine fortgeschrittene Coxarthrose. Eine fokale Nekrose des Stadiums 2 gemäß **ARCO(Association Research Circulation Osseous)-Klassifikation**

**Figure 9:**
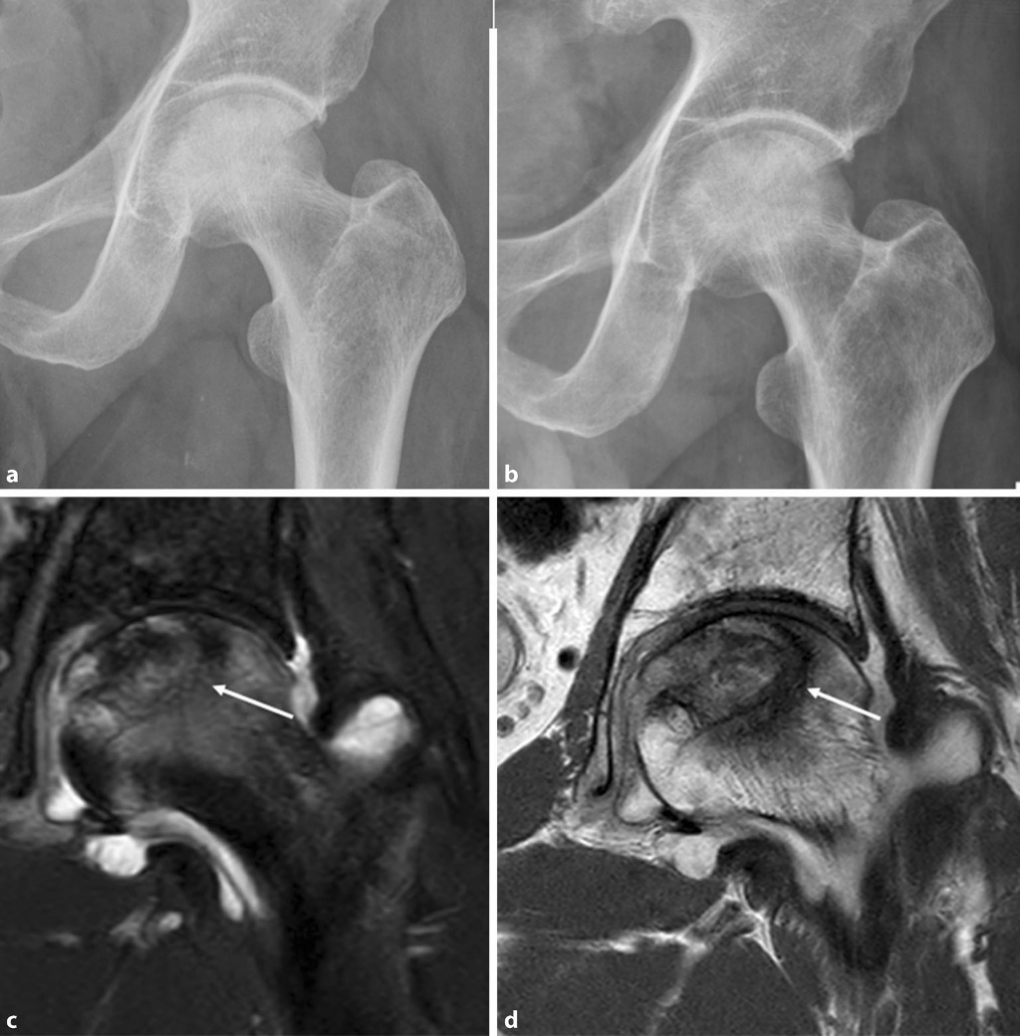

ARCO-Klassifikation

ist irreversibel und in der Projektionsradiographie sichtbar (Abb. 9a, b). In der MRT zeigt sich ein **Doppellinienzeichen**

Doppellinienzeichen

mit KM-Anreicherung des T2-signalreichen Granulationssaums und einer sklerotischen signalarmen Begrenzung (Abb. 9c, d). Das Nekroseareal nimmt kein KM auf. Im Stadium 3 kommt es zu einer subchondralen Fraktur bei allerdings noch erhaltener Gelenkfläche (**„crescent sign“**

„Crescent sign“).

Durch die Darstellung des subchondralen KMÖ im reversiblen Frühstadium ist die MRT das sensitivste bildgebende Verfahren. Im Gegensatz zum transienten KMÖ-Syndrom (Abb. 8b) ist das KMÖ eher subchondral lokalisiert (Abb. 9c). Im Frühstadium können die avaskulären Nekrosen konservativ behandelt werden. Bei Versagen der medikamentösen Therapie kann die **Knochenmarkbohrung**

Knochenmarkbohrung

zielführend sein, da sie den intraossär erhöhten Druck reduziert. Im Endstadium der AVN ist der **Gelenkersatz**

**Figure 10:**
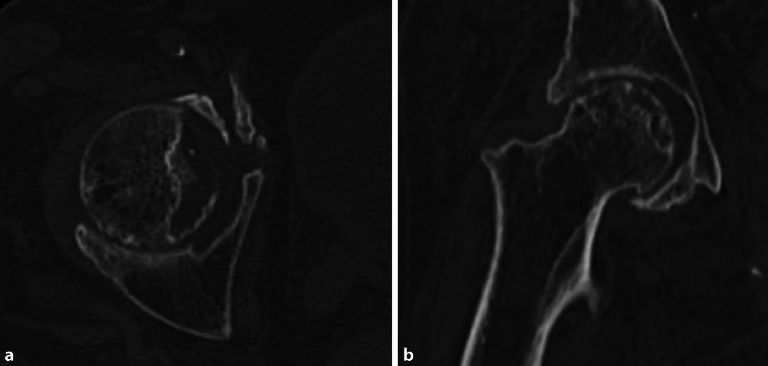

Gelenkersatz

die Therapie der Wahl. Die CT stellt in hoher Auflösung die subchondralen Frakturen und den Einbruch des Femurkopfes im fortgeschrittenen Stadium dar (Abb. 10a, b).

### Merke

Das KMÖ ist bei der Coxarthrose und bei der AVN subchondral im Femurkopf lokalisiert, während das KMÖ beim transienten KMÖ-Syndrom bis in den Schenkelhals hineinreicht.

## Chondroblastom

**Figure 11:**
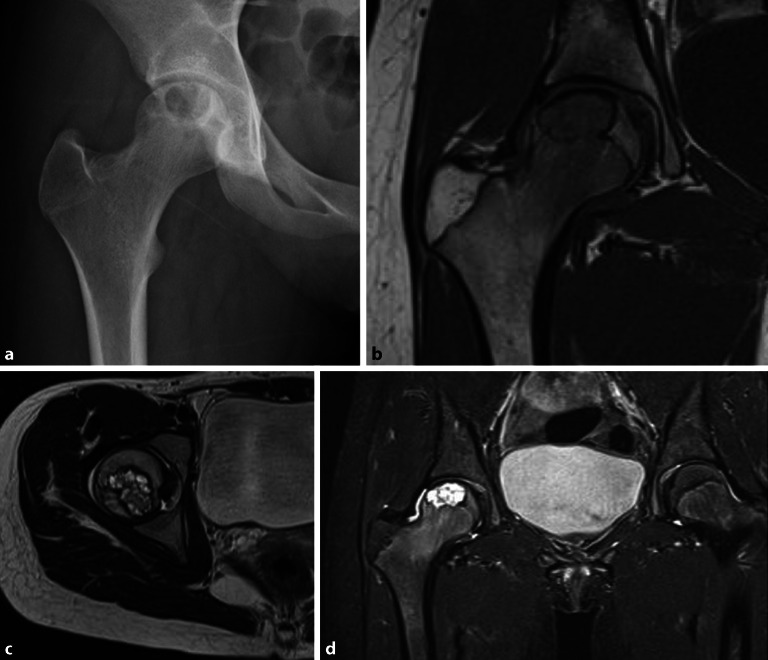

Der gutartige chondrogene Tumor, das Chondroblastom, kommt klassischerweise nur in der Epiphyse oder in der Apophyse bei jungen Patienten vor [12]. 95 % der Tumoren entstehen im Alter zwischen 5 und 25 Jahren mit einem Peak zwischen 10 und 20 Jahren. In manchen Fällen kann sich eine sekundäre aneurysmatische Knochenzyste entwickeln, und vereinzelt wurde sogar eine maligne Transformation mit Gefäßinvasion und Metastasen beobachtet. In der Projektionsradiographie zeigt sich eine lytische Läsion mit Verkalkungen der Matrix (Abb. 11a). In 30 % der Fälle liegt ein Gelenkerguss vor. In der MRT zeigt sich die Läsion mit intermediärem T1-gewichteten (Abb. 11b) und intermediärem bis hohem T2-gewichteten Signal (Abb. 11c) mit variablen signalarmen Arealen, die den Verkalkungen entsprechen. In den protonengewichteten Sequenzen oder in der STIR(„short tau inversion recovery“)-Sequenz kann das Chondroblastom von KMÖ umgeben sein (Abb. 11d). Die **chirurgische Kürettage**

Chirurgische Kürettage,

ggf. mit Knochenaufbau, ist die primäre Therapie für das Chondroblastom.

## Osteoidosteom

**Figure 12:**
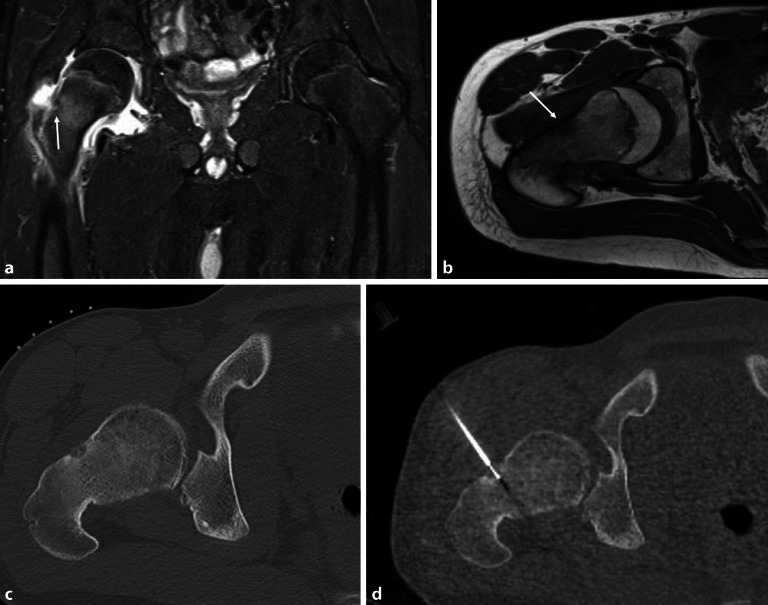

Das Osteoidosteom ist ein gutartiger, schmerzhafter, langsam wachsender Knochentumor der Knochenkortikalis und kommt bei Kindern und Jugendlichen vor. Die Patienten klagen über Nachtschmerz, der sich unter NSAID („non-steroidal antiinflammatory drugs“) deutlich lindert. Der zentrale Nidus besteht histologisch aus einem verkalkten Netz von Knochentrabekeln mit Osteoblasten. Bei der extraartikulären Lage des Osteoidosteoms zeigen sich eine kortikale Verdickung und eine assoziierte Sklerose. Das intraartikuläre Osteoidosteom der Hüfte verursacht einen Erguss und eine Synovitis und kann eine entzündliche Arthritis vortäuschen, da meistens nur eine minimale kortikale Sklerose vorliegt (Abb. 1 und 12a, b). In der MRT ist der Nidus häufig nur schwer zu erkennen. In diesen Fällen ist die CT weiterführend, um den sklerosierenden Nidus hochauflösend darzustellen und die Diagnose zu bestätigen (Abb. 12c). Die **Radiofrequenzablation**

Radiofrequenzablation

ist die Therapie der Wahl, die unter CT-Kontrolle durchgeführt wird (Abb. 12d). Falls dies nicht erfolgreich ist, wird eine chirurgische Sanierung angestrebt.

## Zusammenfassung

Die Kenntnis der unterschiedlichen entzündlichen Erkrankungen der Hüfte ist notwendig, um diese von den Differenzialdiagnosen mit ähnlichem Erscheinungsbild abzugrenzen. Das subchondrale KMÖ ist ein typischer Befund bei der Hüftentzündung, kann allerdings auch bei der aktivierten Osteoarthrose, bei der Osteonekrose oder beim transienten KMÖ-Syndrom bzw. bei Tumoren oder Infektionen vorkommen. Die entzündlich bedingte Synovitis ist schwer von der PVNS oder von der septischen Arthritis zu differenzieren, und bestimmte MRT-Sequenzen (Gradientenechosequenzen) sind hilfreich. Häufig sind mehrere Bildgebungsmodalitäten notwendig, um eine definitive Diagnose zu stellen.

## Fazit für die Praxis

- Eine frühzeitige Kontrolle der Knochendichte bei rheumatischen Patienten, insbesondere unter Kortisontherapie, ist von großer Bedeutung, um rechtzeitig Komplikationen wie eine avaskuläre Osteonekrose und osteoporotische Frakturen zu vermeiden.
- Bei Verdacht auf ein Osteoidosteom ist die Computertomographie zielführend, um den Nidus darzustellen.
- Eine Synovitis der Hüfte kann neben der entzündlich-rheumatologischen Ursache auch durch eine septische Arthritis, ein Osteoidosteom, eine pigmentierte villonoduläre Synovitis oder eine Coxarthrose bedingt sein.

## CME-Fragebogen

Welche Erkrankung zeigt eine subchondrale Sklerose?

Coxarthrose

Rheumatoide Arthritis

Transientes Knochenmarködemsyndrom

Chondroblastom

Synoviale Chondromatose

Was versteht man unter dem Penumbra-Zeichen beim Brodie-Abszess?

Lytische ovaläre Läsion in der Projektionsradiographie

Lytische Läsion mit sklerotischer Randbegrenzung in der Computertomographie (CT)

Periphere Kontrastmittelanreicherung in der CT und in der Magnetresonanztomographie (MRT)

Perifokales Knochenmarködem in der MRT

T2-hyperintenser Granulationssaum in der MRT

Bei welcher Erkrankung ist die diffuse Knorpelbelagverschmälerung der Hüfte zu sehen?

Osteoarthrose

Rheumatoide Arthritis

Chondroblastom

Avaskuläre Osteonekrose

Transientes Knochenmarködem

Welches Kriterium unterscheidet die primäre von der sekundären Form der Chondromatose im Erscheinungsbild?

Lage

Anzahl

Erguss

Synovitis

Größe und Form

Das Doppellinienzeichen der avaskulären Nekrose beschreibt welches Phänomen?

Subchondrale Fraktur

Keilförmiges Knochenmarködem am Schenkelhals

Subchondrale Minderperfusion mit Sklerose und Granulationssaum

Avulsionsfraktur des Trochantor minor

Subchondrale Fraktur des Sakroiliakalgelenks und des ipsilateralen Femurkopfes

Wie wird die Diagnose des transienten Knochenmarködemsyndroms gestellt?

Keimnachweis im Aspirat

In der Verlaufskontrolle durch Abnahme des Ödems

Osteopenische Knochenstruktur in der Projektionsradiographie

Unterbrechung der Kortikalis in der Sonographie

Hotspot in der Szintigraphie

Mit welcher Pathologie kann ein Chondroblastom assoziiert sein?

Enchondrom

Aseptische Nekrose

Osteoidosteom

Aneurysmatische Knochenzyste

Solitäre Knochenzyste

Ein zentraler Nidus ist bei welchem Tumor zu sehen?

Osteoidosteom

Osteosarkom

Weichteilsarkom

Lipom

Knochenzyste

Mit welcher Technik lässt sich die Knorpeldarstellung des Hüftgelenks in der Magnetresonanztomographie (MRT) verbessern?

Niederfeld-MRT

Innenrotation des Beins

Gradientenechosequenzen

Traktions-MR-Arthrographie

Intravenöse Kontrastmittelgabe

Wodurch unterscheidet sich die pigmentierte villonoduläre Synovialitis von der fokalen Synovitis in der Magnetresonanztomographie?

Lokalisation

Ergussmenge

Dicke der Synovialmembran

Form

Erosionen und „blooming“
